# Identification of Fungus-Derived Natural Products as New Antigiardial Scaffolds

**DOI:** 10.1128/spectrum.00647-23

**Published:** 2023-04-11

**Authors:** Matthew A. Hulverson, Samantha A. Michaels, Jin Woo Lee, Karen L. Wendt, Linh T. Tran, Ryan Choi, Wesley C. Van Voorhis, Robert H. Cichewicz, Kayode K. Ojo

**Affiliations:** a Department of Medicine, Division of Allergy and Infectious Diseases, Center for Emerging and Reemerging Infectious Diseases (CERID), University of Washington, Seattle, Washington, USA; b College of Pharmacy, Duksung Women’s University, Seoul, Republic of Korea; c Natural Products Discovery Group, Institute for Natural Products Applications and Research Technologies, Department of Chemistry and Biochemistry, Stephenson Life Sciences Research Center, University of Oklahoma, Norman, Oklahoma, USA; University of Iowa Hospitals and Clinics

**Keywords:** *Giardia lamblia*, antigiardial, fungal extracts, pure natural compounds

## Abstract

There is an unmet need for effective therapies for treating diseases associated with the intestinal parasite Giardia lamblia. In this study, a library of chemically validated purified natural products and fungal extracts was screened for chemical scaffolds that can inhibit the growth of G. lamblia. The phenotypic screen led to the identification of several previously unreported classes of natural product inhibitors that block the growth of G. lamblia. Hits from phenotypic screens of these naturally derived compounds are likely to possess a variety of mechanisms of action not associated with clinically used nitroimidazole and thiazolide compounds. They may therefore be effective against current drug-resistant parasite strains.

**IMPORTANCE** There is a direct link between widespread prevalence of clinical giardiasis and poverty. This may be one of the reasons why giardiasis is a significant contributor to diarrheal morbidity, stunting, and death of children in resource-limited communities around the world. FDA-approved treatments for giardiasis include metronidazole, related nitroimidazole drugs, and albendazole. However, a substantial number of clinical infections are resistant to these treatments. The depth of the challenge is partly exacerbated by a lack of investment in the discovery and development of novel agents for treatment of giardiasis. Applicable interventions must include new drug development strategies that will result in the identification of effective therapeutics, particularly those that are inexpensive and can be quickly advanced to clinical uses, such as products from nature. This study identified novel chemical scaffolds from fungi that can form the basis of future medicinal chemistry optimization of novel antigiardial agents.

## OBSERVATION

The intestinal pathogen Giardia lamblia causes morbidity and mortality on a global scale (1). Unfortunately, higher prevalence of clinical giardiasis correlates with lower socioeconomic status, thus inflicting a disproportionate burden on the young, especially in resource-limited countries (1, 2). Available treatments are limited, cause side effects, and have an increasing risk of drug resistance, which sets the stage for a greater public health concern (3–5). Furthermore, there are limited prospects for additional treatment options due to a lack of investment in the drug development pipelines. These facts emphasize the need for new drug development strategies to discover effective therapeutics, particularly those that can be quickly moved to clinical use. Bioactive compounds from nature have been optimized by evolution to perform specific biological functions without impairing the producing organism’s physiological proficiencies (6, 7). This extrapolative safety profile potentially reduces the cost for clinical development, especially for some antimicrobial agents.

A significant number of clinically used antimicrobial agents are chemically optimized derivatives of natural products (8). Artemisinin and ivermectin are examples of antiparasitic agents derived from natural products (9, 10). Naturally derived molecules are an important, yet relatively underexplored resource for new bioactive chemical scaffolds. The World Health Organization (WHO) has suggested the application of natural products for health care needs since they are widely used, inexpensive, and considered mostly safe (11, 12). We report here a phenotypic screening of a natural product library with the aim of identifying compounds that can be optimized as potent antigiardial treatments.

**FIG 1:**
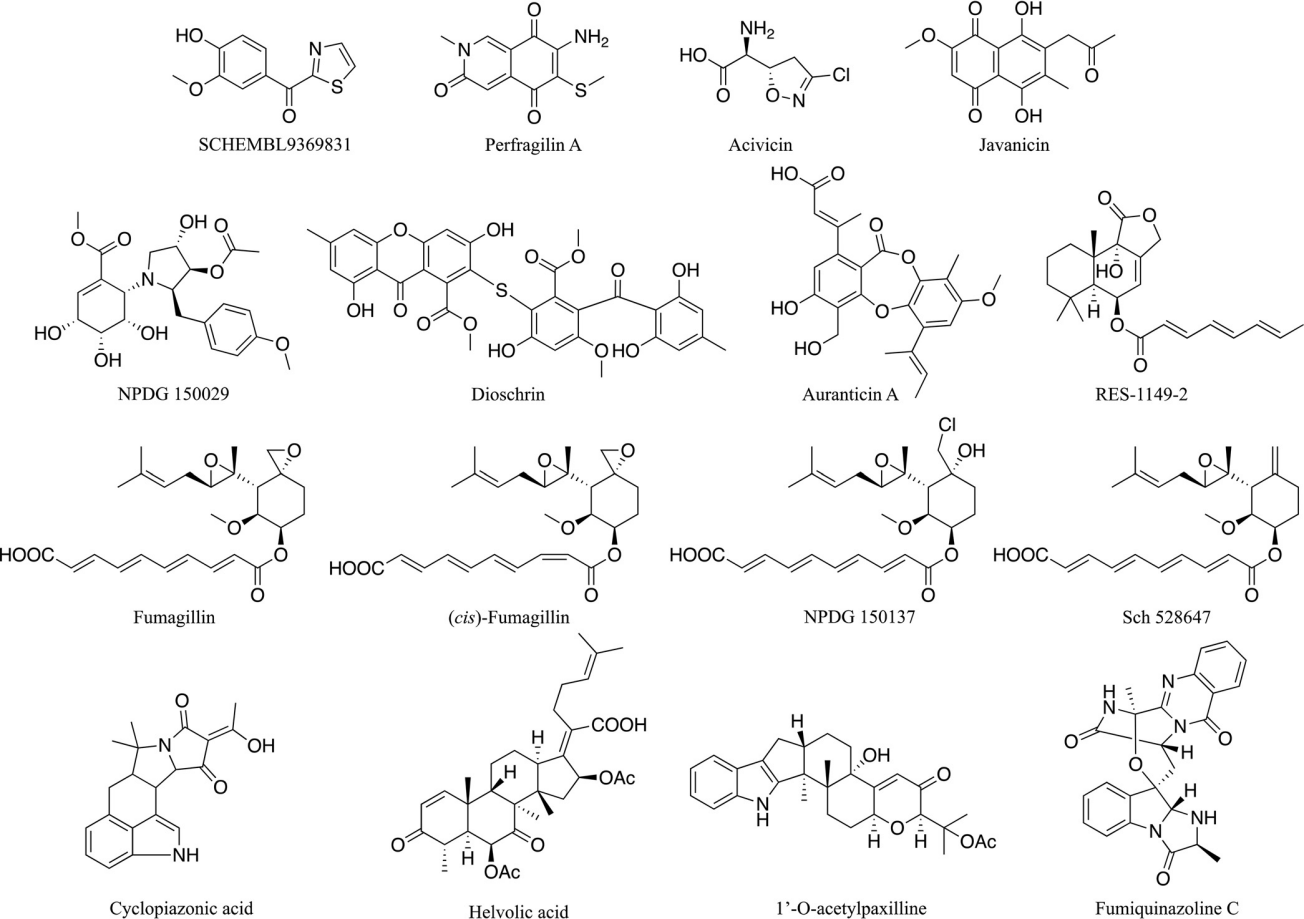
Chemical structures of pure natural products with potent and selective antigiardial activity.

For *in vitro* Giardia assays, we used a click beetle green (CBG99) luciferase-based reporter system that was developed for quantitative bioluminescence readouts of growth in trophozoites (13–15). The CBG99 reporter gene is under the control of the glutamate dehydrogenase promoter, which is highly expressed in the G. lamblia trophozoite stage, making it useful for monitoring growth even in mouse infection models (15). The compound library was obtained from the Natural Products Discovery Group (NPDG) at the University of Oklahoma (16). A combination of 755 purified natural products (pNPs) and 1,056 fungal extracts were screened against G. lamblia strain CBG99. Hits were defined as samples that caused ≥50% growth inhibition activity at 10 μM (pNPs) or 5 μg/mL (extracts). Analysis of the pNP inhibition data showed 38 compounds with ≥50% inhibition of G. lamblia growth and proliferation. Only 25 of the 38 compounds were further analyzed, since 13 showed signs of cytotoxicity in a counterscreen employing human ileocecal adenocarcinoma cells (HCT-8; ATCC, Manassas, VA) tested at 10 μM. Of the noncytotoxic compounds, 16 had ≥75% inhibition of G. lamblia trophozoite growth, with one showing ≥90% inhibition. A summary of the screening data is presented in Table 1. The concentration at which compounds inhibited 50% of G. lamblia trophozoite growth *in vitro* (EC_50_) was determined for the 16 noncytotoxic hits with ≥75% inhibition of G. lamblia trophozoite growth in the primary screening. Only 2 were considered false positives (EC_50_ > 10 μM) (Table 2). The chemical structures of the 16 pNPs are shown in Fig. 1. Four compounds, fumagillin, *cis*-fumagillin, RES-1149-2, and NPDG 150137, had EC_50_ values of <200 nM against G. lamblia trophozoite and were >25 times more potent than metronidazole, which has an EC_50_ of 5 μM (17). As noted, HCT-8 growth and proliferation were not inhibited at 10 μM by those compounds determined to be noncytotoxic, demonstrating that a good safety index could be achieved.

**TABLE 1:** Screening of fungal extracts and pure natural compounds against G. lamblia

Compound type	Inhibition (%)	Total no. of hits	No. of compounds excluded due to cytotoxicity	No. of remaining hits (% of total)
Fungal extracts^*a*^	>90	18	5	13 (1.2)
	75–90	28	2	26 (2.5)
Pure natural compounds^*b*^	>90	7	6	1 (0.1)
	75–90	19	4	15 (2.0)

a Fungal extracts (*n* = 1,056) were screened at 5 μg/mL.

b pNP compounds (*n* = 755) were screened at 10 μM.

**TABLE 2:** pNP hits against G. lamblia^*a*^

NPDG ID	Synonym	% inhibition at 10 μM	EC_50_ ± SD (μM)
120014	SCHEMBL9369831	79	7.11 ± 9.12
120038	Perfraglin A	75	>10
120071	Acivicin	85	>10
150029	Anisomycin 1-*N*-pericosine	86	3.32 ± 0.01
150049	*cis*-Fumagillin	85	0.03 ± 0.02
150051	Dioschrin	82	3.21 ± 0.61
150113	1′-*O*-acetylpaxilline	82	3.40
150125	RES-1149-2	88	0.18 ± 0.11
150134	Auranticin A	89	4.44 ± 3.02
150137		90	0.006
150138	Sch 528647	88	3.09 ± 0.16
160012	Javanicin	89	1.26 ± 1.01
170027	Cyclopiazonic acid	81	1.30 ± 0.71
170028	Helvolic acid	83	3.36 ± 0.14
170035	Fumagillin	88	0.004
170098	Fumiquinazoline C	82	6.92 ± 8.5

a None of the listed pNPs showed any effect on HCT-8 cells at 10 μM.

Testing of the extract library against G. lamblia CBG99 identified 87 extracts with ≥50% inhibition, with 8 of those samples also causing HCT-8 cytotoxicity. Further analysis generated a refined list of 39 noncytotoxic extracts exhibiting ≥75% inhibition of cell growth, among which 13 showed ≥90% inhibition, and 4 of these extracts had ≥95% inhibition. The most potent, noncytotoxic fungal extracts included samples prepared from Pochonia chlamydosporia (with 84% inhibition of Giardia growth), *Cytospora* sp. (95% inhibition), Scedosporium apiospermum (94%), Penicillium chlamydosporia (95%), Cladosporium cladosporioides (91%), *Coniothyrium* sp. (91%), Aspergillus fumigatus (95%), Penicillium janczewskii (85%), Penicillium oxalicum (95%), and Penicillium camemberti (92%). None of the listed crude fungal extracts inhibited HCT-8 cells at 40 μg/mL. Since the dearth of antigiardial drug development is associated with limited available resources (13), it is critical that extracts selected represent the best starting points for identifying additional natural product scaffolds with antiparasitic activity and reduced host toxicity, to provide the shortest path to clinical application. The mammalian cytotoxicity of crude extracts was therefore included as a selection criterion, in addition to EC_50_ values against G. lamblia.

**FIG 2:**
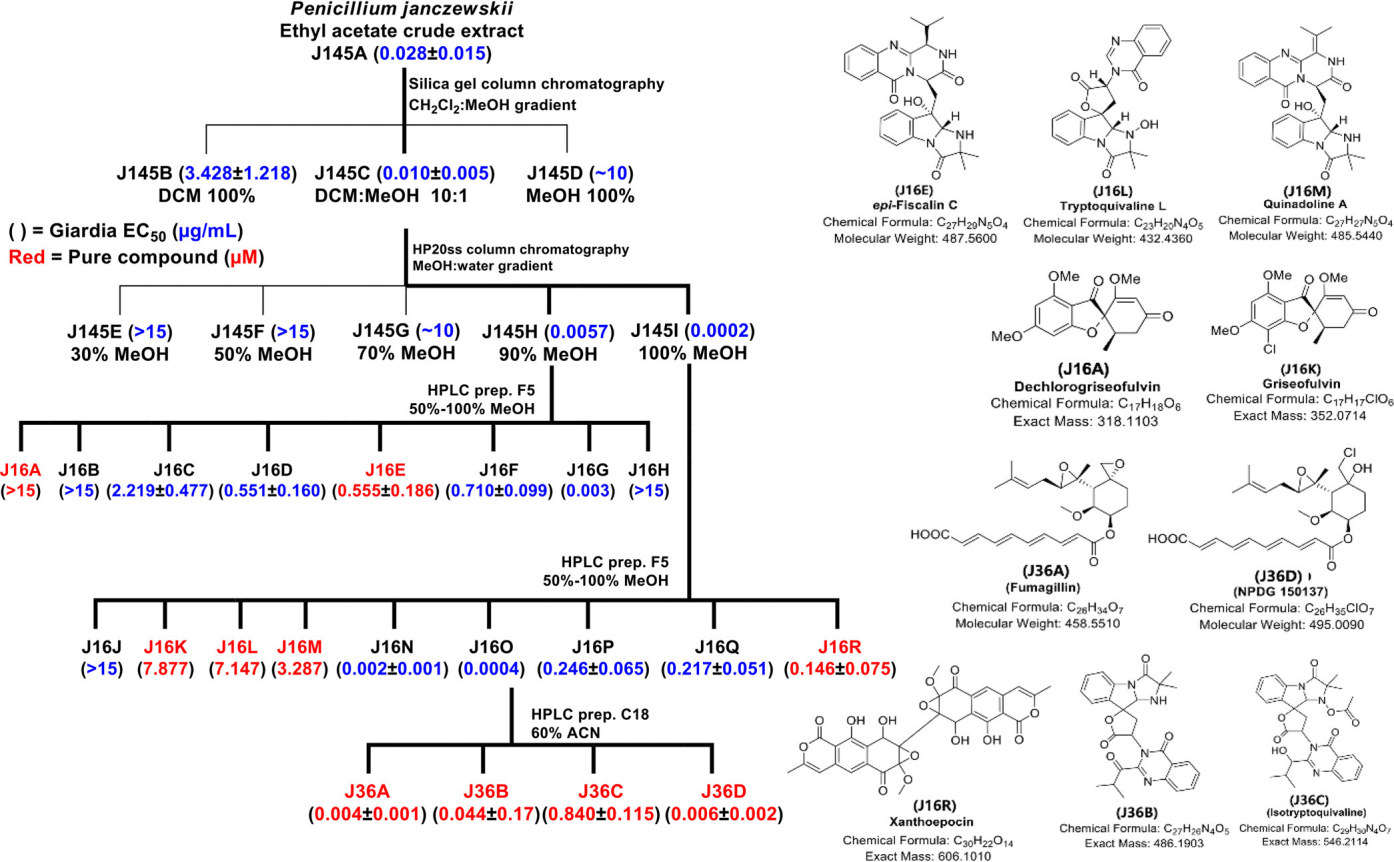
(Left) Crude extract of *P. janczewskii* metabolite dereplication process/solvents and efficacy of the subfractions/pure compounds (EC_50_) against G. lamblia strain CBG99. Identified pure compounds are delineated in red letters. The EC_50_ value of each fraction/pure compound is shown in parentheses. For cytotoxicity assays against the crude extract (J145A) and its subfractions with mammalian cells (on CRL-8155 and HepG2), cytotoxicity was determined to be absent at >40 μg/mL. (Right) Chemical structures of the identified pure metabolites.

Extracts from cultures of *Penicillium janczewskii* were selected based on availability, ease of regrowth, and historical references (18, 19). The observed EC_50_ value of regrown *P. janczewskii* crude extract was 0.13 ng/mL, thereby validating its antigiardiasis activity. *P. janczewskii* is a common filamentous fungus found in nature. The *P. janczewskii* metabolite, griseofulvin, has been shown to be of particular therapeutic value in the treatment of human skin mycoses (19, 20). Further characterization of the antigiardiasis activity and cytotoxicity of *P*. *janczewskii* crude extract (J145A) was carried out to find agents with no toxic signals (Fig. 2). J145A and its 3 antigiardial fractions did not inhibit the growth or proliferation of HepG2 and CRL-8155 cells at 40 μg/mL (21).

Fractionation and dereplication (identification of compounds responsible for the bioactivity of an extract) processes yielded 10 pure compounds with resolved chemical structures (22). All the purified compounds, with the exception of dechlorogriseofulvin (J16A), had various levels of antigiardial effects at the test concentration. The most potent purified compounds from J145A were fumagillin and its analog *cis*-fumagillin (Fig. 2). Fumagillin (EC_50_ = 0.004 μM) and its other potent analogs, *cis*-fumagillin (EC_50_ = 0.030 μM), RES-1149-2 (EC_50_ = 0.180 μM), and NPDG 150137 (EC_50_ = 0.006 μM), provide some structural activity relationship (SAR) data for further optimization of the pharmaceutical potential of the scaffold (Fig. 1 and 2, Table 2). NPDG 150137 is a chlorinated fumagillin derivative that has never been reported; hence, it currently has no trivial name. Three indole alkaloids, tryptoquivaline L (J16L; EC_50_ = 7.147 μM), *epi*-fiscalin C (J16E; EC_50_ = 0.555 μM), and quinadoline A (J16M; EC_50_ = 3.287 μM), as well as xanthoepocin (J16R; EC_50_ = 0.146 μM), isotryptoquivaline (J36C; EC_50_ = 0.840 μM), and its analog (J36B; EC_50_ = 0.044 μM) (Fig. 2), are other potent compounds belonging to chemical scaffolds other than fumagillin. Fumagillin and griseofulvin (J16K; EC_50_ = 7.877 μM) were previously discovered in a high-throughput screening (HTS) of diverse compound libraries as inhibitors of G. lamblia growth (23–25). The reliability of the selection criteria and dereplication processes for a successful antigiardial drug discovery project was validated with the purification of fumagillin, fumagillin analogs, and griseofulvin from crude J145A, as presented in Fig. 2.

Highly potent inhibitors of G. lamblia can be obtained from natural products and fungal extracts, thereby expanding the diversity of sources and chemical spaces available for the discovery of new antigiardial therapeutic agents. Validation of the antigiardial effect of some of the active pNPs was provided by the observed inhibition of G. lamblia growth by other compounds with the same core chemical scaffolds (Fig. 1 and 2). The new chemical scaffolds would be a basis for accelerated development of highly specific antigiardial drugs that utilize new mechanisms of action. Since the new scaffolds discovered in this study are different from the nitroimidazole and thiazolide classes of compounds, there is the possibility that the potential new drugs from the series will be effective against metronidazole-resistant strains. Many of the compounds in this library are not available from commercial sources and were identified as bioactive entities while conducting bioassay-driven discovery projects. Examples include NPDG-150029 and NPDG-150137 (Table 2). Inactive compounds in the extract help to define the SAR of scaffolds. For instance, dechlorogriseofulvin J16A, which is inactive at 15 μg/mL (Fig. 2), helps define likely SAR for griseofulvin analogs. This combination of factors makes the collection especially useful as a unique first-stage screening tool and a highly valuable resource for drug discovery.

### Data availability.

Data will be made available upon request for peer review.

## References

[1] Platts-Mills JA, Babji S, Bodhidatta L, Gratz J, Haque R, Havt A, McCormick BJ, McGrath M, Olortegui MP, Samie A, Shakoor S, Mondal D, Lima IF, Hariraju D, Rayamajhi BB, Qureshi S, Kabir F, Yori PP, Mufamadi B, Amour C, Carreon JD, Richard SA, Lang D, Bessong P, Mduma E, Ahmed T, Lima AA, Mason CJ, Zaidi AK, Bhutta ZA, Kosek M, Guerrant RL, Gottlieb M, Miller M, Kang G, Houpt ER, MAL-ED Network Investigators. 2015. Pathogen-specific burdens of community diarrhoea in developing countries: a multisite birth cohort study (MAL-ED). Lancet Glob Health 3:e564-75. doi:10.1016/S2214-109X(15)00151-5. 26202075 PMC7328884

[2] Donowitz JR, Alam M, Kabir M, Ma JZ, Nazib F, Platts-Mills JA, Bartelt LA, Haque R, Petri WA, Jr. 2016. A prospective longitudinal cohort to investigate the effects of early life giardiasis on growth and all cause diarrhea. Clin Infect Dis 63:792–797. doi:10.1093/cid/ciw391. 27313261 PMC4996141

[3] Nabarro LE, Lever RA, Armstrong M, Chiodini PL. 2015. Increased incidence of nitroimidazole-refractory giardiasis at the Hospital for Tropical Diseases, London: 2008–2013. Clin Microbiol Infect 21:791–796. doi:10.1016/j.cmi.2015.04.019. 25975511

[4] Tejman-Yarden N, Eckmann L. 2011. New approaches to the treatment of giardiasis. Curr Opin Infect Dis 24:451–456. doi:10.1097/QCO.0b013e32834ad401. 21857510

[5] Lalle M. 2010. Giardiasis in the post genomic era: treatment, drug resistance and novel therapeutic perspectives. Infect Disord Drug Targets 10:283–294. doi:10.2174/187152610791591610. 20429863

[6] Atanasov AG, Waltenberger B, Pferschy-Wenzig EM, Linder T, Wawrosch C, Uhrin P, Temml V, Wang L, Schwaiger S, Heiss EH, Rollinger JM, Schuster D, Breuss JM, Bochkov V, Mihovilovic MD, Kopp B, Bauer R, Dirsch VM, Stuppner H. 2015. Discovery and resupply of pharmacologically active plant-derived natural products: a review. Biotechnol Adv 33:1582–1614. doi:10.1016/j.biotechadv.2015.08.001. 26281720 PMC4748402

[7] Atanasov AG, Zotchev SB, Dirsch VM, Supuran CT, International Natural Product Sciences Taskforce. 2021. Natural products in drug discovery: advances and opportunities. Nat Rev Drug Discov 20:200–216. doi:10.1038/s41573-020-00114-z. 33510482 PMC7841765

[8] Newman DJ, Cragg GM. 2012. Natural products as sources of new drugs over the 30 years from 1981 to 2010. J Nat Prod 75:311–335. doi:10.1021/np200906s. 22316239 PMC3721181

[9] Tu Y. 2016. Artemisinin—a gift from traditional Chinese medicine to the world (Nobel lecture). Angew Chem Int Ed Engl 55:10210–10226. doi:10.1002/anie.201601967. 27488942

[10] Shen B. 2015. A new golden age of natural products drug discovery. Cell 163:1297–1300. doi:10.1016/j.cell.2015.11.031. 26638061 PMC5070666

[11] World Health Organization. 2009. Monographs on selected medicinal plants, vol 4. World Health Organization, Geneva, Switzerland.

[12] Anquez-Traxler C. 2011. The legal and regulatory framework of herbal medicinal products in the European Union: a focus on the traditional herbal medicines category. Drug Inf J 45:15–23. doi:10.1177/009286151104500102.

[13] Michaels SA, Hennessey KM, Paragas N, Paredez AR, Ojo KK. 2021. A curious case for development of kinase inhibitors as antigiardiasis treatments using advanced drug techniques. ACS Infect Dis 7:943–947. doi:10.1021/acsinfecdis.0c00919. 33534539

[14] Michaels SA, Hulverson MA, Whitman GR, Tran LT, Choi R, Fan E, McNamara CW, Love MS, Ojo KK. 2022. Repurposing the kinase inhibitor mavelertinib for giardiasis therapy. Antimicrob Agents Chemother 66:e0001722. doi:10.1128/aac.00017-22. 35703552 PMC9295539

[15] Michaels SA, Shih H-W, Zhang B, Navaluna ED, Zhang Z, Ranade RM, Gillespie JR, Merritt EA, Fan E, Buckner FS, Paredez AR, Ojo KK. 2020. Methionyl-tRNA synthetase inhibitor has potent in vivo activity in a novel Giardia lamblia luciferase murine infection model. J Antimicrob Chemother 75:1218–1227. doi:10.1093/jac/dkz567. 32011682 PMC7869794

[16] Lee JW, Collins JE, Wendt KL, Chakrabarti D, Cichewicz RH. 2021. Leveraging peptaibol biosynthetic promiscuity for next-generation antiplasmodial therapeutics. J Nat Prod 84:503–517. doi:10.1021/acs.jnatprod.0c01370. 33565879 PMC7941592

[17] Ranade RM, Zhang Z, Gillespie JR, Shibata S, Verlinde CL, Hol WG, Fan E, Buckner FS. 2015. Inhibitors of methionyl-tRNA synthetase have potent activity against Giardia intestinalis trophozoites. Antimicrob Agents Chemother 59:7128–7131. doi:10.1128/AAC.01573-15. 26324270 PMC4604383

[18] Schmeda-Hirschmann G, Hormazabal E, Rodriguez JA, Theoduloz C. 2008. Cycloaspeptide A and pseurotin A from the endophytic fungus Penicillium janczewskii. Z Naturforsch C J Biosci 63:383–388. doi:10.1515/znc-2008-5-612. 18669024

[19] Curtis P, Grove J. 1947. A fungistatic and bacteriostatic red pigment produced by a strain of the Penicillium nigricans-janczewskii series. Nature 160:574–575. doi:10.1038/160574b0. 20340768

[20] Grove J, McGowan J. 1947. Identity of griseofulvin and “curling-factor.” Nature 160:574. doi:10.1038/160574a0. 20269865

[21] Vidadala RSR, Ojo KK, Johnson SM, Zhang Z, Leonard SE, Mitra A, Choi R, Reid MC, Keyloun KR, Fox AMW, Kennedy M, Silver-Brace T, Hume JCC, Kappe S, Verlinde CLMJ, Fan E, Merritt EA, Van Voorhis WC, Maly DJ. 2014. Development of potent and selective Plasmodium falciparum calcium-dependent protein kinase 4 (PfCDPK4) inhibitors that block the transmission of malaria to mosquitoes. Eur J Med Chem 74:562–573. doi:10.1016/j.ejmech.2013.12.048. 24531197 PMC4024383

[22] Du L, Robles AJ, King JB, Powell DR, Miller AN, Mooberry SL, Cichewicz RH. 2014. Crowdsourcing natural products discovery to access uncharted dimensions of fungal metabolite diversity. Angew Chem Int Ed Engl 53:804–809. doi:10.1002/anie.201306549. 24285637 PMC4028707

[23] Chen CZ, Kulakova L, Southall N, Marugan JJ, Galkin A, Austin CP, Herzberg O, Zheng W. 2011. High-throughput Giardia lamblia viability assay using bioluminescent ATP content measurements. Antimicrob Agents Chemother 55:667–675. doi:10.1128/AAC.00618-10. 21078930 PMC3028786

[24] Kulakova L, Galkin A, Chen CZ, Southall N, Marugan JJ, Zheng W, Herzberg O. 2014. Discovery of novel antigiardiasis drug candidates. Antimicrob Agents Chemother 58:7303–7311. doi:10.1128/AAC.03834-14. 25267663 PMC4249522

[25] Bonilla-Santiago R, Wu ZJ, Zhang LH, Widmer G. 2008. Identification of growth inhibiting compounds in a Giardia lamblia high-throughput screen. Mol Biochem Parasitol 162:149–154. doi:10.1016/j.molbiopara.2008.08.005. 18796315 PMC2597095

